# Metal accumulation by sunflower (*Helianthus annuus L*.) and the efficacy of its biomass in enzymatic saccharification

**DOI:** 10.1371/journal.pone.0175845

**Published:** 2017-04-24

**Authors:** Saurabh Sudha Dhiman, Xin Zhao, Jinglin Li, Dongwook Kim, Vipin C. Kalia, In-Won Kim, Jae Young Kim, Jung-Kul Lee

**Affiliations:** 1 Department of Chemical Engineering, Konkuk University, Seoul, Republic of Korea; 2 Department of Civil and Environmental Engineering, Seoul National University, Seoul, Republic of Korea; 3 Phygen, Daejeon, Republic of Korea; 4 CSIR-IGIB, Delhi University Campus, Mall Road, Delhi, India; Korea University, REPUBLIC OF KOREA

## Abstract

Accumulation of metal contaminants in soil as a result of various industrial and anthropogenic activities has reduced soil fertility significantly. Phytoextraction of metal contaminants can improve soil fertility and provide inexpensive feedstock for biorefineries. We investigated the hyperaccumulation capacity of sunflower (*Helianthus annuus*) biomass by cultivating these plants in various concentrations of metal contaminants. Sunflowers were grown in soils contaminated with various levels of heavy metals (10–2,000 mg/kg dry soil). The degree of metal uptake by different parts of the biomass and the residual concentration in the soil were estimated through inductively coupled plasma mass spectrometry. An almost 2.5-fold hyperaccumulation of Zn^2+^ was observed in the leaf and flower biomass compared with the concentration in the soil. For the subsequent saccharification of biomass with hyperaccumulated contaminants, a fungal lignocellulosic consortium was used. The fungal consortium cocktail retained more than 95% filter paper activity with 100 mM Ni^2+^ ions even after 36 h. The highest saccharification yield (SY, 87.4%) was observed with Ni^2+^ as the contaminant (10 mg/kg dry wt), whereas Pb^2+^ (251.9 mg/kg dry wt) was the strongest inhibitor of biomass hydrolysis, resulting in only a 30% SY. Importantly, the enzyme cocktail produced by the fungal consortium resulted in almost the same SY (%) as that obtained from a combination of commercial cellulase and β-glucosidase. Significant sugar conversion (61.7%) from *H*. *annuus* biomass hydrolysate occurred, resulting in the production of 11.4 g/L of bioethanol. This is the first study to assess the suitability of phytoremediated sunflower biomass for bioethanol production.

## Introduction

Apart from industrial applications of dry sunflower biomass, growing sunflowers (SF) have shown the potential to absorb various metal contaminants such as Ni, Cu, As, Pb, and Cd [1, 2]. Most of the heavy metals are non-biodegradable and toxic to numerous organisms, including humans; therefore, they must be removed from ecosystems [3]. Because growing SF can accumulate high concentrations of metal contaminants, they are considered “hyperaccumulators” of heavy metals [4–6]. Although lignocellulosic material can serve as a feedstock for sustainable biorefinery, sufficient research on phytoextracted hyperaccumulated sunflower biomass has not been conducted. The biggest challenge in conversion of biomass with hyperaccumulated contaminants into fermentable sugars is the inhibition of hydrolytic enzymes by absorbed metal contaminants [7]. Because of the complexity of dry biomass and presence of metal contaminants, it is difficult to hydrolyze a hyperaccumulator efficiently with a single enzyme system [8, 9]. Therefore application of a crude enzyme cocktail may be an efficient strategy to hydrolyze metal-contaminated biomass [10].

Crude lignocellulosic enzyme cocktails extracted from single fungal strain, i.e., *Armillaria gemina* and *Pholiota adiposa*, isolated in-house facilitate efficient hydrolysis of non-contaminated biomass [11, 12]. In the current study, a lignocellulosic cocktail obtained from a fungal consortium of *P*. *adiposa* and *A*. *gemina* was used for the saccharification of hyperaccumulated *Helianthus annuus* (sunflower) biomass. The concentrations of several metal contaminants (As, Cd, Co, Cr, Cu, Fe, Mn, Ni, Pb, and Zn) in soil were calculated, and the quantities of those metals that had been accumulated in different parts of the biomass were determined. The suitability of the saccharification hydrolysate obtained from the biomass was also tested in a coupled fermentation reaction. This is the first study to assess the suitability of phytoremediated *H*. *annuus* biomass for bioethanol production.

## Materials and methods

### *H*. *annuus*: Cultivation, front-end processing, and characterization

Sunflower plants were cultivated in PVC pots (30 cm × 26 cm) filled with 11 kg (dry weight) of test soil, with three replications for each concentration of heavy metal. Cultivation was carried out in a greenhouse at 22°C and pH 6.5–7.5 for 75 days with a photoperiod of nearly 12 h [13, 14]. The heavy metal concentrations of the soil ranged up to100 mg/kg for As, 100 mg/kg for Cd, 450 mg/kg for Cu, 100 mg/kg for Ni, 2,000 mg/kg for Pb, and 300 mg/kg for Zn. For analysis of plants, the SF in the pots were carefully removed from the soils; the roots (R_sf_), stems (S_sf_), leaves (L_sf_), and flowers (F_sf_) were then separated and washed with distilled water to remove soil particles. The roots, stems, leaves, and flowers were dried in an oven (SH-MPM, Vision, Korea) at 105°C for 24 hours, and the dry weight of the plant tissues was measured. Dried plant tissues were homogenized using a mill to measure heavy metal concentrations. The homogenized plant tissues were then digested with a solution of HNO_3_, H_2_O_2_, and distilled H_2_O (9:1:1, v/v/v) as described previously [15]. The digested samples were filtered through 0.45-μm syringe filter and the 25 ml in volumetric flasks completed with the addition of deionized water. The heavy metal concentrations in the plant tissues were determined using inductively coupled plasma mass spectrometry (ICP-MS) [15, 16].

A mechanical shredder was used to cut the various parts (particle size < 5 mm) of the oven-dried (60°C) SF biomass. After shredding, each part of the biomass was stored separately in large polythene bags under dry conditions at 4°C until required. SF seeds were finely ground using an electronic digester, and the resulting fine powder was stored in the conditions mentioned above. Standard NREL protocols were followed to determine the cellulose, hemicellulose (xylan), and lignin contents in the biomass before and after pretreatment [17].

### Fungi: Identification, maintenance, and enzyme production

Fungal strains used in current investigation were previously isolated and identified [12, 18]. Selected strains were deposited in the Korean Culture Center of Microorganisms (KCCM). The KCCM accession numbers for *P*. *adiposa* SKU714 and *A*. *gemina* KJS114 KCCM are 11187P and 11186P, respectively. To produce a lignocellulosic enzyme cocktail showing high catalytic efficiency, an inoculum ratio of 1:2 (w/v) in production medium was adopted for both *P*. *adiposa* and *A*. *gemina* [19]. A new fungus that produced laccase was also isolated, and its ITS1-5.8S-ITS2 rDNA region was amplified using a set of specific primers for phylogenetic analysis.

The fungal consortia were characterized by determining their catalytic activities as described in a previous report [20]: filter paper activity (FPU); the action of β-glucosidase (BGL) on *p*-nitrophenyl-β-d-glucopyranoside (*p*NPG), endo-β-1,4 glucanase (EG) activity, xylanase activity, and the activity of laccase related to the rate of oxidation of 1 mM 2,2-azinobis-[3-ethylbenzothiazoline-6-sulfonate] (ABTS). The dinitrosalicylic acid (DNSA) reagent method was used to estimate the release of reducing sugars (RS) [21]. The residual FPU of the crude consortium enzyme was determined using different concentrations of metal contaminants at 35°C and pH 5.0 under static conditions.

### Pretreatment: The thermochemical method with 2% sodium hydroxide

Alkali-mediated pretreatment of different biomass parts was carried out as described previously [19]. Pretreatment followed extensive washing of the biomass with distilled water until the pH of the effluent reached 7.0 and soaking of the biomass in sodium phosphate buffer solution (pH 5.0) for 14 h. Centrifugation (8000 g; 10 min) was performed to separate the biomass from the buffer solution. The biomass was subsequently dried (60°C; 12 h) to a constant weight.

### Detoxification: Fungal laccase and biomass with hyperaccumulated contaminants

Crude laccase from *Tyromyces chioneus* (TcLac) was used to detoxify the alkali-pretreated SF biomass. The production medium from the *T*. *chioneus* culture contained only activity specific for laccase enzyme, and no manganese peroxidase or lignin peroxidase activity was observed. Physical parameters such as pH, incubation time, and laccase dosage were optimized for the detoxification of pretreated biomass using a conventional “one-variable optimization” approach. Initial detoxification experiments were conducted with 2 g (dry weight) of pretreated rice straw in 100-mL Erlenmeyer flasks mixed with 25 mL of 50 mM buffer (pH 2.5–6.0) at 30°C in under shaking conditions (150 rpm). The Folin–Ciocalteu method was used to determine the concentrations of total phenols present in supernatants in the detoxification experiment [22].

### Microtox^®^: Confirmation of residual metal contaminants

To confirm the retention of metal contaminants in the pretreated and detoxified hyperaccumulated SF biomass before enzymatic saccharification, effluent at the pretreatment stage was used as an indicator. Because TcLac-catalyzed detoxification can cause contaminants to leach from the pretreated substrate, the effluent was also analyzed after the detoxification stage. A fundamental concept underlying the use of effluent is that leaching of metal contaminants significantly increases the ecotoxicity of the effluent. The higher the ecotoxicity, the more prominent the metal leaching; consequently, there is less retention of the metal contaminants in the SF biomass. Effluents released from the pretreatment of non-contaminated SF biomass and non-detoxified biomass were used as controls. The pretreated effluent was analyzed using the 81.9% toxicity test protocol as described previously [23]. The reduction in intensity of light emitted by bioluminescent *Vibrio fischeri* after 5 and 15 min of exposure to metal contaminants was the assay end point. Therefore, EC_*50-5min*_ and EC_*50-15min*_ denote the effective concentrations (EC) of a contaminant that reduced bioluminescence to 50% of its initial value [24, 25]. These EC values were calculated using a standard procedure described previously [23].

### Saccharification: Optimization of parameters

Different physical parameters affecting the saccharification yield (SY) were optimized through a conventional “one-variable optimization” methodology using uncontaminated control biomass. Each physical parameter, i.e., temperature (T), enzyme dose (ED), substrate concentration (SC), incubation time (IT), and rpm, was optimized separately for each part of the SF biomass, i.e., the R_sf_, S_sf_, L_sf_, and F_sf_. To evaluate the effect of each parameter, the yields of RS and SY were calculated using fixed non-optimized conditions (30°C, pH 4.5, and incubation for 48 h using 10 mg protein/g-biomass). To optimize the IT, samples were taken from the reaction mixture at regular intervals (6 h) up to 48 h to estimate the RS (mg/g-substrate) and the SY (%). Similarly, saccharification experiments were conducted over a broad range of temperatures (25–45°C) and rotation speeds (50–250 rpm) for optimization. The ED was optimized using various concentrations of enzymes (ranging from 2.5 to 15 FPU/mg of enzyme. In the case of the SC, different concentrations (0.5–2% w/v) of pretreated substrate were tested. Augmentation of commercial BGL (Novozyme-188) was also investigated using a fixed dose (10 mg protein/g-biomass) of enzyme in combination with commercial cellulase (Celluclast 1.5 L). The RS was estimated using the DNSA assay at 540 nm, and the SY was calculated as described previously [26, 27].

### Bioethanol: Production from hydrolysates of the hyperaccumulator *H*. *annuus*

After enzymatic saccharification, the samples were centrifuged (8000 g; 4°C; 20 min) to separate clear solution (termed saccharification hydrolysate) from the biomass pellet. Saccharification hydrolysates obtained from different parts of the SF biomass were collected, pooled, and concentrated using N_2_ purging for bioethanol production. The saccharification hydrolysate of *H*. *annuus* (CHHA) was concentrated to obtain an RS concentration of 5% (w/v). A liquid seed culture (48-h old) of *Saccharomyces cerevisiae* (ATCC 32167) was used as the inoculum (10% v/v) to convert the concentrated RS to bioethanol. A chemically defined medium (yeast extract 5.0 g/L; (NH_4_)_2_SO_4_ 10.0 g/L; KH_2_PO_4_ 4.5 g/L, and MgSO_4_.7H_2_O 1.0 g/L) was mixed with the concentrated RS solution and inoculated for culture at 30°C for up to 48 h under static conditions. Bioethanol production from the saccharification hydrolysate was compared with that from a synthetic medium containing dextrose as a carbon source. Samples were collected every 6 h, centrifuged (8000 g; 4°C; 20 min), and analyzed for dextrose consumption and ethanol production using the flame ionization detector (FID) of a gas chromatograph (GC). Helium (He) gas was used as the mobile phase, whereas air and hydrogen (H_2_) were used with the FID. The ethanol volumetric productivity (g/L/h), yield of ethanol produced (g/L), and sugar conversion efficiency (%) were calculated as described previously [20].

## Results & discussion

### Hyperaccumulation of metal contaminants

SF are highly adsorbent and have been used to bioaccumulate heavy and toxic metals [14]. In the case of Ni, As, and Cd at low concentrations (10–100 mg/kg dry soil), Cd^2+^ (161 mg/kg dry wt) accumulated to its highest concentration in sunflower leaves and flowers, whereas in the stems only 78.4 mg/kg dry wt was accumulated (Table 1). Similarly, for Ni and As, flowers and leaves showed higher phytoremediation capacity than stems (Table 1). In the case of Zn^2+^, as the initial concentration of the metal ion in the soil increased, its absorption by the plant also increased (Table 1). Nearly 2.5-fold more Zn^2+^ was observed in flowers and leaves compared with the concentration in the soil (i.e., 300 mg-Zn/kg dry soil). The concentration absorbed by the stems alone was nearly 1.5-fold more than the supplied Zn^2+^ (Table 1). When Cu^2+^ was supplied in moderate concentrations (300 and 450 mg/kg dry soil) to the growing *H*. *annuus*, the least accumulation was observed in all plant parts. Only 75.3 mg of Cu^2+^ was present in the leaves and flowers, and only 10.3 mg was present in the stems of the SF (Table 1). With regard to the accumulation of the highest concentrations of Pb (2000 mg/kg dry soil), the stems showed the highest capacity, storing 252 mg of Pb^2+^ compared to the 149 mg accumulated by leaves and flowers. Even at a moderate concentration of Pb (1000 mg/kg dry soil), the stems showed the highest accumulation capacity (136 mg). However, when a lower concentration (250 mg/kg dry soil) of Pb^2+^ was supplied, both stems (29.1 mg) and leaves and flowers (26.3 mg) accumulated the metal at similar concentrations (Table 1). The heavy metal concentrations in sunflower tissues growing in control soil were analyzed and used as a control.

**Table 1:** Saccharification yield obtained with hyperaccumulated biomass.

Metal	Metal (soil)	Biomass	Metal (biomass)	Saccharification yield (%)			
	mg/kg-soil	component	mg/kg-biomass	FC^a^	FC+188^b^	CC^c^	CC+188^d^
Ni	10	Stem	0.5±0.1	87.4±5.6	87.2±7.7	76.4±6.6	82.5±7.7
		L + F*	3.1±0.3	87.1±4.4	86.4±6.6	79.8±6.5	85.6±7.2
	50	Stem	2.5±0.1	86.4±5.6	85.2±8.1	75.9±6.3	83.5±7.3
		L + F*	13.9±1.2	84.7±6.6	82.6±7.1	78.5±6.1	82.4±7.3
	100	Stem	15.6±1.1	84.2±7.1	82.5±6.6	72.5±6.8	79.8±7.1
		L + F*	99.1±8.8	80.3±7.2	74.9±6.3	72.4±6.3	75.7±7.3
As	10	Stem	N.D.	84.7±6.6	85.2±8.1	76.2±6.1	82.6±8.1
		L + F*	N.D.	86.2±7.7	86.8±6.3	74.9±6.6	84.5±8.3
	50	Stem	N.D.	84.7±7.3	86.4±8.2	73.5±6.7	82.1±8.4
		L + F*	10.2±0.9	84.5±7.6	83.2±7.4	72.6±5.8	79.2±7.2
	100	Stem	44.1±2.2	81.2±7.2	79.6±6.3	72.6±5.8	78.6±7.5
		L + F*	43.9±3.4	81.7±6.5	77.6±6.8	72.4±7.1	77.9±7.1
Cd	50	Stem	40.3±3.8	84.2±7.9	78.4±7.2	71.6±7.2	79.5±7.4
		L + F*	98.1±7.7	69.4±5.4	62.9±5.1	56.7±4.5	61.2±5.5
	100	Stem	78.4±6.6	63.3±3.3	76.5±7.2	61.8±5.8	68.2±6.1
		L + F*	161±14	43.6±4.1	36.4±2.8	32.5±2.2	34.6±2.2
Zn	200	Stem	128±12	82.1±6.6	78.1±6.4	69.5±5.5	72.4±6.3
		L + F*	269±23	65.1±5.5	55.3±4.6	49.7±4.2	50.6±4.8
	300	Stem	443±33	42.9±3.3	29.5±1.1	33.4±3.3	34.1±4.1
		L + F*	808±45	27.3±1.1	13.3±1.1	16.9±1.1	17.2±2.1
Cu	300	Stem	14.2±1.1	81.9±3.3	80.1±7.5	70.3±6.8	77.6±6.8
		L + F*	43.4±2.2	76.7±6.6	73.2±6.3	62.9±5.8	70.6±6.9
	450	Stem	10.3±0.9	70.9±5.5	69.8±5.8	61.3±5.9	67.5±6.2
		L + F*	75.3±5.5	66.3±6.6	60.2±5.9	55.6±4.2	62.5±6.1
Pb	250	Stem	29.1±2.1	77.7±6.5	76.8±6.7	68.4±6.3	72.6±5.8
		L + F*	26.3±1.1	80.0±7.1	79.2±7.1	66.5±6.1	75.8±6.3
	1000	Stem	136±24	38.7±3.3	29.6±2.1	29.4±1.2	31.2±2.2
		L + F*	63.2±6.6	54.5±4.4	47.8±5.3	43.6±3.3	48.6±3.3
	2000	Stem	252±29	29.6±1.6	18.4±1.3	17.6±1.2	18.3±1.1
		L + F*	149±22	36.8±3.3	30.5±2.8	22.5±2.1	25.1±2.2
Control		Stem	—	87.2±6.5	87.4±7.4	83.6±8.5	88.1±7.8
		L + F*	—	82.3±6.6	82.9±7.2	81.7±7.4	83.3±7.9

*leaf and flower contents were mixed

^a^enzyme cocktail of fungal consortium

^b^fungal consortium with Novozyme BGL

^c^commercial cellulase

^d^commercial cellulase with Novozyme BGL

Saccharification experiments were conducted at 35^°^C, pH 5.0, RPM 200 and with an enzyme dose of 10 mg protein/g-biomass

### Enzyme production and inhibitory effect of metal ions

Nutrient use by growing microbes is a limiting factor in the co-culture of two species of fungi [20]. Use of wheat bran as a carbon source has resulted in the highest FPU of 2.57 U/mL after “one-variable optimization” of nutritional components. Physical parameters were optimized using a 7-L fermenter (with a 3-L working volume) through this procedure. After optimizing all physical and nutritional parameters, the synergistic proliferation of both selected fungi showed a 2.1–3.0-fold increase in FPU activity compared to that exhibited by either single culture [15, 19]. Different activities and protein contents of Celluclast have been described previously [15].

Because the consortium enzyme was utilized for hydrolysis of the metal-contaminated biomass, the sensitivity of the consortium enzyme to the presence of different metal ions was also investigated (S1 Fig). Only residual FPU activity up to 48 h was calculated, as this was when the fungal consortium enzyme was loaded to determine filter paper activity for saccharification. Among the metal ions tested, the maximal inhibitory effect was shown by Pb^2+^, with even a 1 mM concentration of the ion sufficient to produce residual activity of 40% after 36 h (S1 Fig). Arsenic and cadmium ions also showed significant inhibition, and 40% residual activity was observed after 36 and 48 h, respectively (S1 Fig). The consortium enzyme showed similar resistance to copper and nickel ions, and more than 85% residual activity was observed after 36 h with both metal contaminants (S1 Fig). The consortium enzyme showed the highest resistance to zinc ions and retained more than 91% residual activity with a 100 mM concentration after 36 h at 35°C (S1 Fig). Resistance of crude enzyme to metal contaminants can be attributed to the synergism of different catalytic subunits of the consortium cocktail [28]. TcLac exhibited the *K*_m_ value of 75.5 μM with 2,6-dimethoxypehnol as a substrate, which is lowest K_m_ value ever reported for any fungal laccase. The phylogenetic position of the TcLac-producing newly isolated *T*. *chioneus* fungus (KFCC11573P) is shown in S2 Fig [29]. Crude TcLac was also found to be stable for up to 4 h of incubation with various concentrations of metal ions. Resistance to metal ions was not checked beyond 4 h because this was the time required for the detoxification procedure. A similar trend of lignocellulase inhibition was observed with the inhibition of TcLac activity in the presence of various metal ion contaminants. Maximal inhibition of TcLac activity (10 U/mL) was observed with the highest concentration (100 mM) of Pb^2+^ and Zn^2+^ ions after 4 h of incubation at 37°C with shaking (150 rpm) (S3 Fig). Similarly, 50 and 100 mM concentrations of Cd^2+^ and As^2+^ ions exhibited the same level of enzyme inhibition, resulting in 34.5 and 29.6% residual TcLac activity, respectively (S3 Fig). Among the other metal contaminants, TcLac showed the highest resistance to 100 mM of Cu^2+^ and retained up to 66.3% residual laccase activity after 4 h of incubation (S3 Fig). Moreover, TcLac retained more than 90% residual activity in the presence of each metal contaminant (1 mM), even after 4 h of incubation, suggesting that it is highly suitable for the detoxification of SF biomass that had hyperaccumulated metal contaminants.

### Alkali-mediated pretreatment of biomass with hyperaccumulated contaminants

Thermochemical pretreatment was carried out to remove lignin contents and to make cellulosic material available for the subsequent enzymatic hydrolysis step [30]. For biomass with hyperaccumulated contaminants, thermochemical pretreatment was carried as described previously [12]. A quantitative analysis of the main structural components, i.e., cellulose, hemicellulose, lignin, and ash, was carried out before and after pretreatment according to a standard procedure [19, 31]. After pretreatment, the cellulosic contents increased as a result of the removal of unwanted hemicellulosic and ligneous material from the biomass; their concentrations are shown in S1 Table.

### Enzymatic detoxification of pretreated biomass

**Fig 1:**
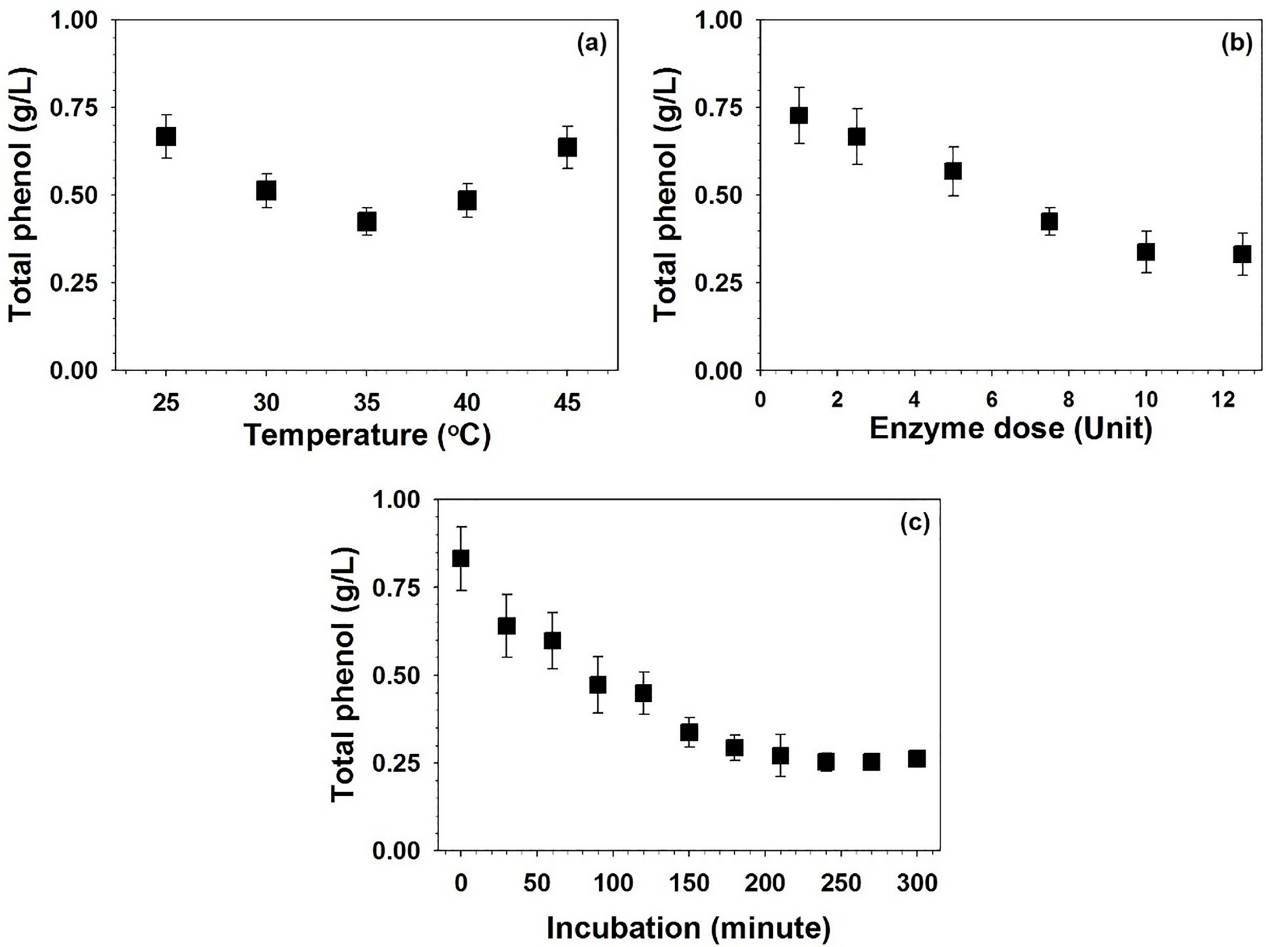
Optimization of the operational parameters for efficient detoxification* of pretreated sunflower biomass using TcLac. **(a) Temperature, (b) enzyme dose, and (c) incubation time.** *The detoxification process was optimized using stems in the biomass.

Residual phenolic compounds (PCs) from the biomass pretreatment stage are known to inhibit hydrolyzing enzymes [32]. Therefore, pretreated of SF biomass was detoxified using TcLac over a broad range of temperatures (from 25 to 45°C) to obtain a saccharification hydrolysate of *H*. *annuus* (CHHA). The highest detoxification efficiency was observed at 35°C (Fig 1A). Any deviation from the optimized temperature resulted in a significant loss of detoxification efficiency (Fig 1A). This might indicate that the optimal catalytic rate of TcLac is achieved at the optimal temperature. A similar trend was observed during optimization of the TcLac dose for detoxification of residual PCs. The optimal (10 U/mL) TcLac dose resulted in 0.33 g/L of total residual PCs (Fig 1B) compared with 0.83 g/L of PCs in the control biomass. A further increase in the TcLac dose did not result in a significant reduction in the PC concentration (Fig 1B). Optimization of the incubation time further decreased the total residual PCs to 0.25 g/L, which was 69.8% less than that from the non-detoxified biomass (Fig 1C). To shorten the detoxification step, IT beyond 240 mins was not considered. A significantly higher dose of fungal laccase (100 U/mL) was used to achieve 77.5% detoxification of sugarcane bagasse after 5 h of incubation [33]. However, the current study utilized only 10 U/mL of TcLac: 1/10th the dosage reported in a previous study [34]. Previous studies have also confirmed the inducing effect of detoxification on ethanol productivity [35]. Laccase-catalyzed detoxification coupled with a synthetic mediator requires a subsequent washing step [36]. To simplify the process, a mediator was not used in this study.

For optimization of temperature, experiments were performed at 35°C and pH 5.0 (maintained by the addition of 0.1 N HCl), using a dose of 5 U/mL TcLac for 180 mins with shaking (150 rpm). For optimization of the enzyme dose, experiments were performed at 35°C and pH 5.0 (maintained by the addition of 0.1 N HCl) for 180 mins with shaking (150 rpm). For optimization of the incubation time, experiments were performed at 35°C and pH 5.0 (maintained by the addition of 0.1 N HCl) using 10 U/mL TcLac with shaking (150 rpm).

### Confirmation of metal hyperaccumulation

The effluent collected from the pretreatment and detoxification stages was subjected to the 81.9% toxicity test protocol. There was no significant difference in acute toxicity of the effluent collected after the pretreatment of biomass with hyperaccumulated contaminants in comparison with that of the control (S2 Table). Marginal increases of 9.8 and 11.9% in acute toxicity were observed in the effluent of biomass with hyperaccumulated contaminants after 5 and 15 mins, respectively, of incubation in the Microtox^®^ assay (S2 Table). This indicates that most metal contaminants were retained in the biomass even after pretreatment. The increase in acute toxicity can be attributed to loosely bound metal ions that leached out because of the harsh thermochemical pretreatment. Moreover, the effluent of the pretreated biomass with hyperaccumulated contaminants showed 66.1 and 72.3% less acute toxicity than the mixed metal contaminant solution (S2 Table), which further promotes the retention of metal contaminants in the biomass. Similarly, the effluent collected after detoxification of the pretreated hyperaccumulated SF biomass had 55.8 and 68.2% less acute toxicity than the mixed metal contaminant solution (S2 Table). This suggests that even after detoxification more than 80% of the metal contaminants are retained by the biomass.

To accurately estimate residual heavy metal concentrations in pretreated biomass samples, the ICP-OES technique was used [16]. In the case of Cd^2+^ contaminants, maximal leaching as a result of pretreatment was observed with leaf biomass samples, where approximately 16.7% of contaminants were released. Similarly, in the case of Pb^2+^ contaminants, the highest leaching (4.81%) of metal ions was observed in leaf-only biomass samples (Table 2). However, in the case of Zn^2+^, a mixture of flower and seed biomass showed the highest leaching (2.88%), which was similar (2.45%) to the contaminant leaching observed from pretreated leaf biomass (Table 2). The high level of leaching of metal contaminants from pretreated leaves and the mixture of flowers and seeds may be a result of their low concentrations of cellulosic and hemicellulosic contents, as these are the primary constituents responsible for retaining nutrients and other materials [37]. Hence, it was confirmed that most of the pretreated samples of biomass retained more than 80% (mg/kg-biomass) of original concentration of metal contaminants as observed through Microtox^®^ (S2 Table).

**Table 2:** Concentrations of various metal contaminants present in the biomass before and after pretreatment.

Biomass part	Metal contaminants (mg/metal-kg)					
	Cd	Pb	Zn	Ni	As	Cu
Metal contaminants before pretreatment (mg/kg-biomass)						
Root	3.21±0.31	17.2±1.5	54.0±6.7	1.93±0.31	ND	20.8±8.3
Stem	2.46±0.61	7.01±2.32	40.1±9.6	ND	ND	7.26±1.67
Leaf	4.84±0.42	29.1±5.9	53.1±4.1	ND	ND	15.0±3.4
Flower & seed	3.57±0.33	10.0±3.8	45.0±4.9	0.22±0.02	ND	17.0±3.6
Metal contaminants after pretreatment (mg/kg-biomass)						
Root	2.94±0.52	16.3±2.3	53.1±6.7	1.77±0.83	ND	18.4±1.3
Stem	2.19±0.29	6.88±0.69	39.6±4.5	ND	ND	6.63±0.85
Leaf	4.03±0.66	27.7±3.6	51.8±6.4	ND	ND	13.8±2.2
Flower & seed	3.21±0.48	9.73±1.12	43.7±2.4	0.17±0.05	ND	15.4±2.4

ND: Not determined

### Enzyme catalyzed saccharification

The main purpose of the current phytoremediation work was to investigate the bioabsorption of metal contaminants and produce materials of commercial interest from biomass with hyperaccumulated contaminants. The suitability of hyperaccumulated *H*. *annuus* biomass was confirmed by enzymatic hydrolysis and the production of sugars through saccharification. Saccharification experiments were conducted at 35°C, pH 5.0, and 200 rpm, with an enzyme dose of 10 mg protein/g-biomass. The presence of 10 and 50 mg of absorbed metal ions (Ni and As) per kg-biomass did not affect the final saccharification yield (SY%). The highest SY (87.4%) was observed in stalks when Ni^2+^ was supplied at 10 mg/kg-soil. Similarly, for phytoremediation of As^2+^, the highest SY (86.2%) was observed with a combination of flowers and leaves. However, when Cd was supplied at 50 mg/kg-soil, significant bioaccumulation was observed in all parts of the biomass. This confirms the high phytoremediation capacity of *H*. *annuus* for Cd, which was responsible for a slight decrease in the final SY% compared to the yield obtained with bioaccumulation of similar concentrations of Ni and As (Table 1). Of Ni, As, and Cd at 100 mg/kg-soil, the highest and lowest SYs were observed when phytoremediation was carried out with Ni^2+^ (84.2%) and Cd^2+^ (63.3%). In the case of phytoremediation of Zn^2+^ metal, the highest SY (82.1%) was observed when 128 mg of metal was accumulated in *H*. *annuus* stalks. Even in the presence of 2.5-fold higher Zn^2+^ concentrations than that in the soil, nearly 28% SY was observed. This confirmed the metal tolerance of the produced fungal consortium (Table 1). However, a high concentration (> 250 mg/kg-soil) of Pb was detrimental to the catalytic behavior of the fungal consortium. The highest (80%) SY was achieved when Pb^2+^ was absorbed at a moderate concentration (26.3 mg) by *H*. *annuus* stalks. As the concentration of absorbed Pb^2+^ increased in the different parts of the biomass, the final SY decreased correspondingly (Table 1). This confirmed the detrimental effect of Pb^2+^ on the catalytic action of the fungal consortium.

A significant saccharification yield of 87.4% was obtained using 10 mg protein/g-biomass of the fungal consortium cocktail with 5.5% (w/v) substrate loading. To our knowledge, this is the first report on saccharification of metal-adsorbed sunflower biomass used for phytoremediation. A comparison in terms of the SY in the current study with that of previous reports is summarized in S3 Table [38–42]. For an evaluation of the SY efficiency of the consortium cocktails, a mixture of commercial Celluclast (10 mg protein/g-biomass) and Novozyme BGL (6 mg protein/g-biomass) was used as a control.

### Production of bioethanol using saccharification hydrolysate

The suitability of saccharification hydrolysates obtained from the hyperaccumulator *H*. *annuus* was confirmed by the production of bioethanol. A similar saccharification hydrolysate was produced by stalks and a mixture of leaves and flowers; therefore, the fermentation reaction was coupled with only one of these hydrolysates. The saccharification hydrolysate from stalks was selected because, after detoxification, the residual lignin in the stalks was less (~7%) than that in the mixture of flowers and leaves. Moreover, the final SY% obtained from the stalks (Ni^2+^; 10 mg/kg-soil; 0.5 mg/kg-biomass) was higher than the yield obtained from the mixture of flowers and leaves. The final ethanol yield obtained from the hydrolysate was 11.4 (g/L), corresponding to 61.7% sugar conversion efficiency (Table 3). The remaining sugar content was low (14 g/L), which confirmed the consumption of a significant amount of sugar and indicated the efficiency of the process, as well as the suitability of saccharification hydrolysates of *H*. *annuus* for biofuel production. In the case of contaminant-free sunflower biomass, ethanol concentrations up to 21 g/L were achieved using a high temperature pretreatment (220°C) step, followed by solid state fermentation [43]. A few other reports of the use of sunflower biomass for saccharification have indicated ethanol production up to 27.88 g/L [44]. However, in the case of biomass contaminated with other metals, viz., sorghum, 17.1 mg of ethanol/ g-solid content was obtained [45]. Hence, results of this study confirm the suitability of hyperaccumulated sunflower biomass for biofuel production, as the concentrations of ethanol obtained from metal-contaminated and non-contaminated biomass were comparable.

**Table 3:** Parameters defining the fermentation of saccharification hydrolysates obtained from phytoremediated *Helianthus annuus* with *Saccharomyces cerevisiae*.

Kinetic parameters	Hydrolysate media^a^
Initial sugar (g/L)^b^	50.0±5.5
Residual sugar (g/L)	13.8±2.2
Sugar consumption (%)	72.4±7.7
Ethanol (g/L)	11.4±1.3
Ratio of ethanol yield to consumed sugars	0.31±0.06
Ethanol volumetric productivity (g/L/h)	0.31±0.05
Efficiency of sugar conversion to ethanol (%)	61.7±6.6

^a^Hydrolysate media made-up of reducing sugars released after enzymatic hydrolysis

^b^A mixture of 93% dextrose, 5% xylose, and <2% other sugars

## Conclusions

We have reported the use of a consortium enzyme from *P*. *adiposa* and *A*. *gemina* for efficient hydrolysis of *H*. *annuus* biomass. A conventional “one-parameter standardization” approach was used to maximize the yield of RS. A saccharification yield of 87.3%, corresponding to 534 mg/g-substrate of RS was obtained. Activity of the fungal consortium enzyme was on par with that of commercial enzymes for the saccharification of *H*. *annuus* biomass. The SF accumulated 2.5-fold more metal (i.e., Zn) in their biomass than was present in the soil. Metal contamination in the soil did not significantly inhibit the final SY and subsequent fermentation, thereby indicating the suitability of SF for phytoremediation and subsequent saccharification for bioethanol production.

## Supporting information

S1 Fig **Inhibition of fungal consortium lignocellulases in the presence of different metal contaminants: (a) As, (b) Cd, (c) Pb, (d) Cu, (e) Ni, and (f) Zn ions.** The enzyme was incubated at 37°C for (●) 12 h, (○) 24 h, (▼) 36 h, and (Δ) 48 h under specific conditions.
(DOCX)

S2 Fig A dendrogram of *Tyromyces chioneus* and its related strains. (DOCX)

S3 Fig **Residual laccase activity of *Tyromyces chioneus* with different concentrations of (a) As; (b) Cd; (c) Pb; (d) Cu; (e) Ni; and (f) Zn ions.** The enzyme was incubated at 37°C for (●) 1 h, (○) 2 h, (▼) 3 h, and (Δ) 4 h under specific conditions.
(DOCX)

S1 Table Composition of different parts of the sunflower biomass before and after alkali pretreatment. (DOCX)

S2 Table Evaluation of the acute toxicity of effluent samples determined using a Microtox analyzer and represented by the average EC_50_* values. *EC_50-*5min*_: Inhibition in bioluminescence after 5 min of incubation; EC_50-*15min*_: Inhibition in bioluminescence after 15 min of incubation. ^a^effluent collected after pretreatment of the non-contaminated biomass; ^b^effluent collected after pretreatment of the metal-contaminated biomass; ^c^effluent of non-detoxified pretreated biomass; ^d^effluent of detoxified pretreated biomass; ^e^solution containing 250 μM of each metal contaminant used in the current study.
(DOCX)

S3 Table Comparative analysis of saccharification yield (%) obtained from pretreated sunflower biomass. (DOCX)
