# Correction: Metal accumulation by sunflower (*Helianthus annuus L*.) and the efficacy of its biomass in enzymatic saccharification

**DOI:** 10.1371/journal.pone.0179746

**Published:** 2017-06-09

**Authors:** Saurabh Sudha Dhiman, Xin Zhao, Jinglin Li, Dongwook Kim, Vipin C. Kalia, In-Won Kim, Jae Young Kim, Jung-Kul Lee

There are errors in the Funding section. The correct funding information is as follows: This paper was supported by Konkuk University in 2013.
