# Supplementary figures and images for: Metal accumulation by sunflower (Helianthus annuus L.) and the efficacy of its biomass in enzymatic saccharification
Source: PLoS One. 2017 Apr 24;12(4):e0175845. doi: 10.1371/journal.pone.0175845 (PMC5402931; doi:10.1371/journal.pone.0175845)

**S1 Fig.**


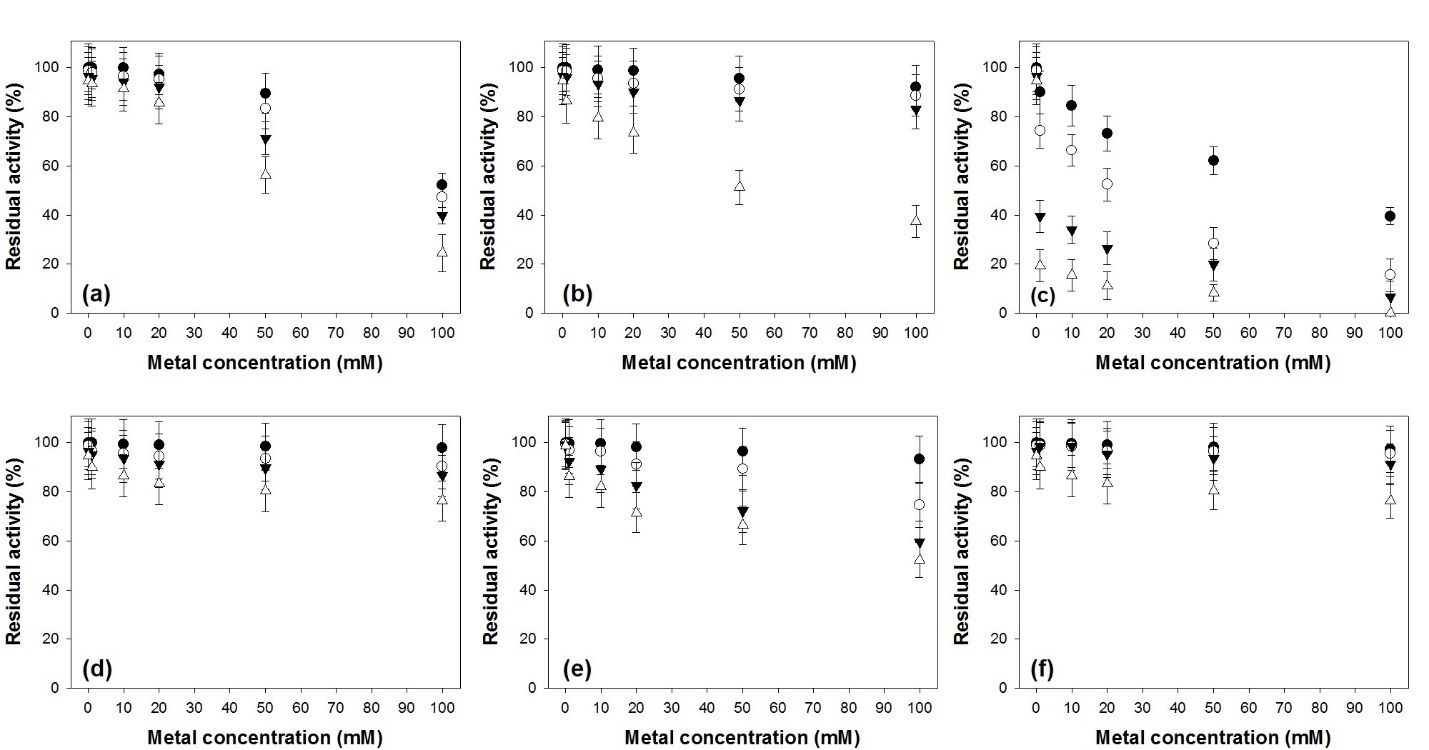

Supplement: S1 Fig — Inhibition of fungal consortium lignocellulases in the presence of different metal contaminants: (a) As, (b) Cd, (c) Pb, (d) Cu, (e) Ni, and (f) Zn ions. The enzyme was incubated at 37°C for (●) 12 h, (○) 24 h, (▼) 36 h, and (Δ) 48 h under specific conditions. (DOCX) [file pone.0175845.s001.docx]

**S2 Fig.**


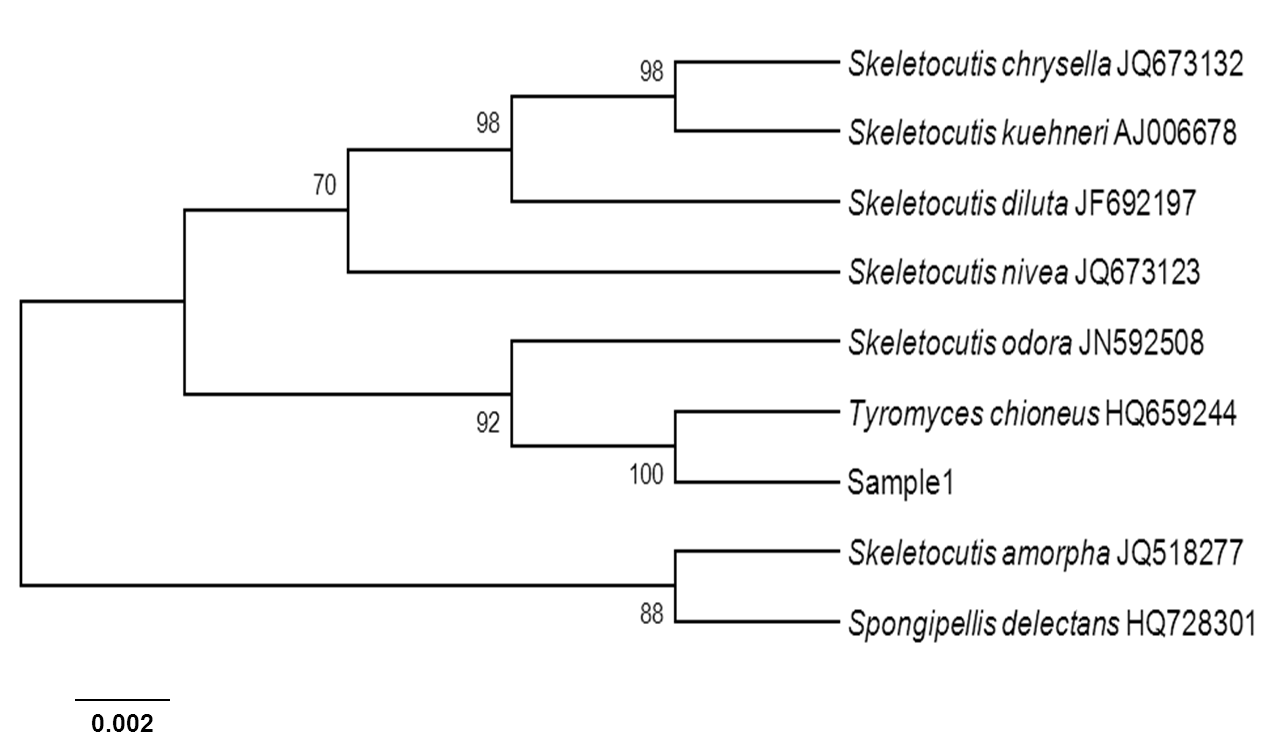

Supplement: S2 Fig — (DOCX) [file pone.0175845.s002.docx]

**S3 Fig.**


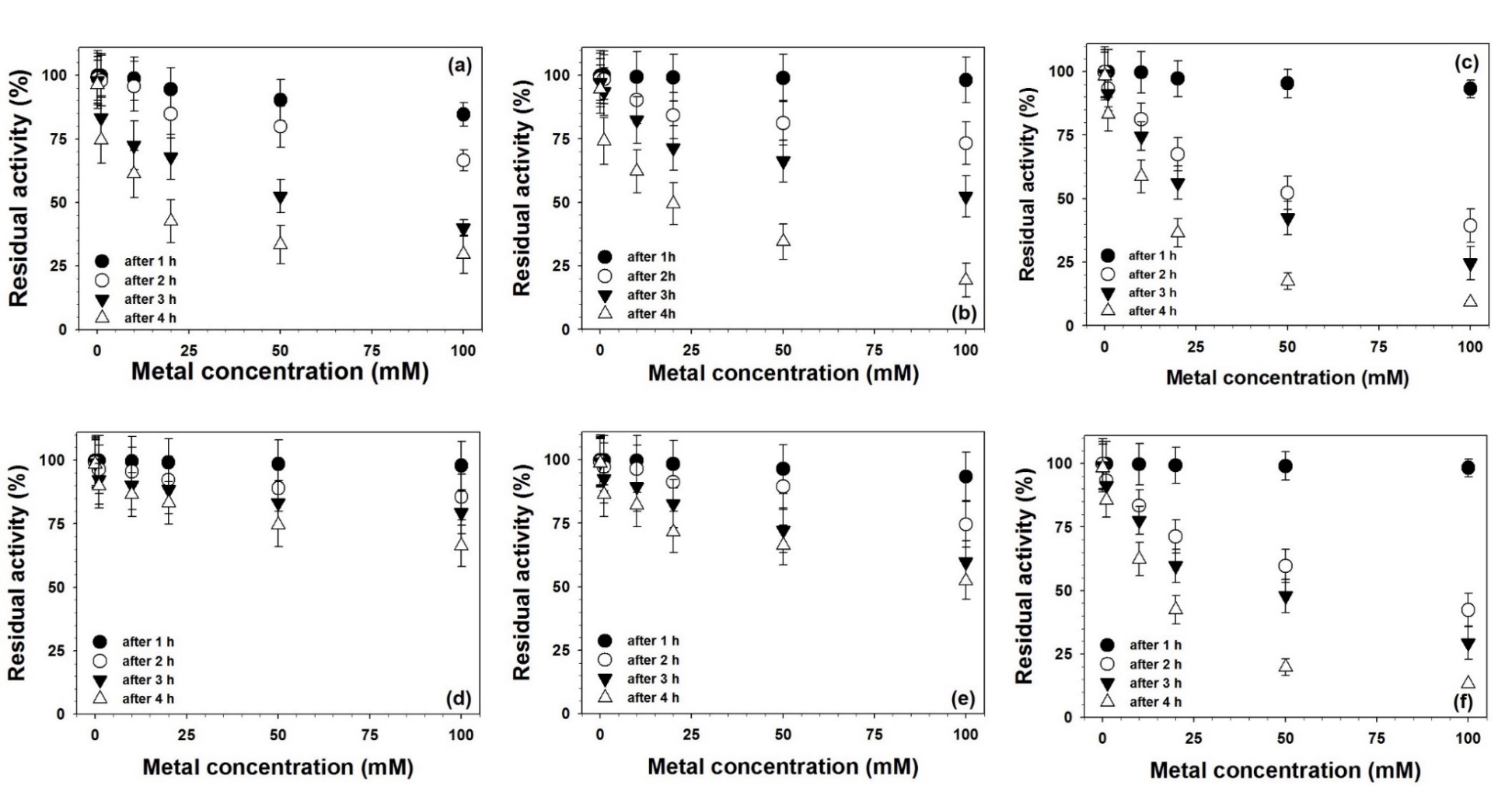

Supplement: S3 Fig — Residual laccase activity of Tyromyces chioneus with different concentrations of (a) As; (b) Cd; (c) Pb; (d) Cu; (e) Ni; and (f) Zn ions. The enzyme was incubated at 37°C for (●) 1 h, (○) 2 h, (▼) 3 h, and (Δ) 4 h under specific conditions. (DOCX) [file pone.0175845.s003.docx]
