# Supplementary material for: Metal accumulation by sunflower (Helianthus annuus L.) and the efficacy of its biomass in enzymatic saccharification
Source: PLoS One. 2017 Apr 24;12(4):e0175845. doi: 10.1371/journal.pone.0175845 (PMC5402931; doi:10.1371/journal.pone.0175845)
Supplement: S1 Table — (DOCX) [file pone.0175845.s004.docx]

**S1 Table. Composition of different parts of the sunflower biomass before and after alkali pretreatment.**

| Biomass part | Cellulose (% w/w) | Hemicellulose (% w/w) | Lignin (% w/w) |
| --- | --- | --- | --- |
| *before pretreatment* | | | |
| Root | 18.8±2.3 | 12.7±3.5 | 32.7±4.5 |
| Stem | 34.9±4.2 | 20.1±3.2 | 29.0±3.4 |
| Leaf | 11.8±2.6 | 13.4±2.1 | 28.6±3.6 |
| Flower & seed | 8.98±0.95 | 4.26±0.82 | 13.6±2.3 |
| *after pretreatment* | | | |
| Root | 12.7±2.5 | 2.56±0.72 | 5.24±1.14 |
| Stem | 24.3±4.9 | 4.47±1.22 | 7.33±1.42 |
| Leaf | 8.44±1.7 | 1.82±0.36 | 2.44±0.48 |
| Flower & seed | 6.19±1.3 | 0.43±0.08 | 0.74±0.14 |
