# Supplementary material for: Metal accumulation by sunflower (Helianthus annuus L.) and the efficacy of its biomass in enzymatic saccharification
Source: PLoS One. 2017 Apr 24;12(4):e0175845. doi: 10.1371/journal.pone.0175845 (PMC5402931; doi:10.1371/journal.pone.0175845)
Supplement: S2 Table — *EC50-5min: Inhibition in bioluminescence after 5 min of incubation; EC50-15min: Inhibition in bioluminescence after 15 min of incubation. aeffluent collected after pretreatment of the non-contaminated biomass; beffluent collected after pretreatment of the metal-contaminated biomass; ceffluent of non-detoxified pretreated biomass; deffluent of detoxified pretreated biomass; esolution containing 250 μM of each metal contaminant used in the current study. (DOCX) [file pone.0175845.s005.docx]

**S2 Table. Evaluation of the acute toxicity of effluent samples determined using a Microtox analyzer and represented by the average EC_50_* values.**

| Sample | Parameter | EC_50_ (%) |
| --- | --- | --- |
| Control effluent – I^a^ | EC_50-_*_5 min_* | 51 ± 8 |
|  | EC_50-_*_15 min_* | 42 ± 9 |
| Test effluent – I^b^ | EC_50-_*_5 min_* | 56 ± 7 |
|  | EC_50-_*_15 min_* | 47 ± 8 |
| Control effluent – II^c^ | EC_50-_*_5 min_* | 38 ± 7 |
|  | EC_50-_*_15 min_* | 33 ± 7 |
| Test effluent – II^d^ | EC_50-_*_5 min_* | 43 ± 6 |
|  | EC_50-_*_15 min_* | 41 ± 7 |
| Mixed contaminant | EC_50-_*_5 min_* | 19 ± 6 |
| solution^e^ | EC_50-_*_15 min_* | 13 ± 6 |

*EC_50-_*_5min_*: Inhibition in bioluminescence after 5 min of incubation; EC_50-_*_15min_*: Inhibition in bioluminescence after 15 min of incubation

^a^effluent collected after pretreatment of the non-contaminated biomass; ^b^effluent collected after pretreatment of the metal-contaminated biomass; ^c^effluent of non-detoxified pretreated biomass; ^d^effluent of detoxified pretreated biomass; ^e^solution containing 250 µM of each metal contaminant used in the current study
