# Supplementary material for: Metal accumulation by sunflower (Helianthus annuus L.) and the efficacy of its biomass in enzymatic saccharification
Source: PLoS One. 2017 Apr 24;12(4):e0175845. doi: 10.1371/journal.pone.0175845 (PMC5402931; doi:10.1371/journal.pone.0175845)
Supplement: S3 Table — (DOCX) [file pone.0175845.s006.docx]

**S3 Table.** **Comparative analysis of saccharification yield (%) obtained from pretreated sunflower biomass.**

| Pretreatment condition | Enzyme source and units | Sunflower stalk (%) | Saccharification yield (%) | Reference |
| --- | --- | --- | --- | --- |
| Steam explosion for 1.5h with 0.5% NaOH | *T. reesei* Rut-C 30 cellulase  (25 FPU/g) | 5 | 57.8 | (38) |
| Steam explosion for 1.5h with 0.5% NaOH | *T. reesei* Rut-C 30 cellulase  (25 FPU/g) | 5 | 59.8 | (39) |
| Sulfuric acid (0.02 M) overnight steam exploded at 207 °C and 21 kg/cm^2^ for 3 minutes | Celluclast  2.67% (w/w) | 6 | 11.9 | (40) |
| Pre-treatment at 220◦C steam explosion | Celluclast: Novozyme BGL  15 FPU:12.5U/g | 10 | 72 | (41) |
| Hydrothermal treatment  180^o^C for 30 min | 8 FPU | 5 | 67 | (42) |
| Thermochemical  (210^o^C for 60 mins) | 10 FPU/mg | 5.5 | 87 | This study |
